# Using regulatory genomics data to interpret the function of disease variants and prioritise genes from expression studies

**DOI:** 10.12688/f1000research.13577.2

**Published:** 2018-02-23

**Authors:** Enrico Ferrero

**Affiliations:** 1 Computational Biology, GSK, Medicines Research Centre, Stevenage, SG1 2NY, UK

**Keywords:** bioconductor, r, rstats, regulatory genomics, functional genomics, genetics, gwas, transcriptomics, integration, multiomics

## Abstract

The identification of therapeutic targets is a critical step in the research and developement of new drugs, with several drug discovery programmes failing because of a weak linkage between target and disease.

Genome-wide association studies and large-scale gene expression experiments are providing insights into the biology of several common diseases, but the complexity of transcriptional regulation mechanisms often limits our understanding of how genetic variation can influence changes in gene expression. Several initiatives in the field of regulatory genomics are aiming to close this gap by systematically identifying and cataloguing regulatory elements such as promoters and enhacers across different tissues and cell types.

In this Bioconductor workflow, we will explore how different types of regulatory genomic data can be used for the functional interpretation of disease-associated variants and for the prioritisation of gene lists from gene expression experiments.

## Introduction

Discovering and bringing new drugs to the market is a long, expensive and inefficient process ^1, 2^. The majority of drug discovery programmes fail for efficacy reasons ^3^, with up to 40% of these failures due to lack of a clear link between the target and the disease under investigation ^4^. Target selection, the first step in drug discovery programmes, is thus a critical decision point. It has previously been shown that therapeutic targets with a genetic link to the disease under investigation are more likely to progress through the drug discovery pipeline, suggesting that genetics can be used as a tool to prioritise and validate drug targets in early discovery ^5, 6^.

One of the biggest challenges in translating findings from genome-wide association studies (GWASs) to therapies is that the great majority of single nucleotide polymorphisms (SNPs) associated with disease are found in non-coding regions of the genome, and therefore cannot be easily linked to a target gene ^7^. Many of these SNPs could be regulatory variants, affecting the expression of nearby or distal genes by interfering with the transcriptional process ^8^.

The most established way to map disease-associated regulatory variants to target genes is to use expression quantitative trait loci (eQTLs) ^9^, variants that affect the expression of specific genes. The GTEx consortium profiled eQTLs across 44 human tissues by performing a large-scale mapping of genome-wide correlations between genetic variants and gene expression ^10^. However, depending on the power of the study, it might not be possible to detect all existing regulatory variants as eQTLs. An alternative is to use information on the location of promoters and distal enhancers across the genome and link these regulatory elements to their target genes. Large, multi-centre initiatives such as ENCODE ^11^, Roadmap Epigenomics ^12^ and BLUEPRINT ^13, 14^ mapped regulatory elements in the genome by profiling a number of chromatin features, including DNase hypersensitive sites (DHSs), several types of histone marks and binding of chromatin-associated proteins in a large number of cells and tissues. Similarly, the FANTOM consortium used cap analysis of gene expression (CAGE) to identify promoters and enhancers across hundreds of cells and tissues ^15^.

Knowing that a certain stretch of DNA is an enhancer is however not informative of the target gene(s). One way to infer links between enhancers and promoters *in silico* is to identify significant correlations across a large panel of cell types, an approach that was used for distal and promoter DHSs ^16^ as well as for CAGE-defined promoters and enhancers ^17^. Experimental methods to assay interactions between regulatory elements also exist. Chromatin interaction analysis by paired-end tag sequencing (ChIA-PET) ^18, 19^ couples chromatin immunoprecipitation with DNA ligation to identify DNA regions interacting thanks to the binding of a specific protein. Promoter capture Hi-C ^20, 21^ extends chromatin conformation capture by using "baits" to enrich for promoter interactions and increase resolution.

Overall, linking genetic variants to their candidate target genes is not straightforward, not only because of the complexity of the human genome and transcriptional regulation, but also because of the variety of data types and approaches that can be used. To address this problem, we developed STOPGAP, a database of disease variants mapped to their most likely target gene(s) using several different types of regulatory genomic data ^22^. The database is currently undergoing a major overhaul and will eventually be superseded by POSTGAP. A valid and recent alternative is INFERNO ^23^, though it does only rely on eQTL data for target gene assignment. These resources implement some or all of the approaches that will be reviewed in the workflow and constitute good entry points for identifying the most likely target gene(s) of regulatory SNPs. However, as they tend to hide much of the complexity involved in the process, we will not use them and rely on the original datasets instead.

**Figure 1.**
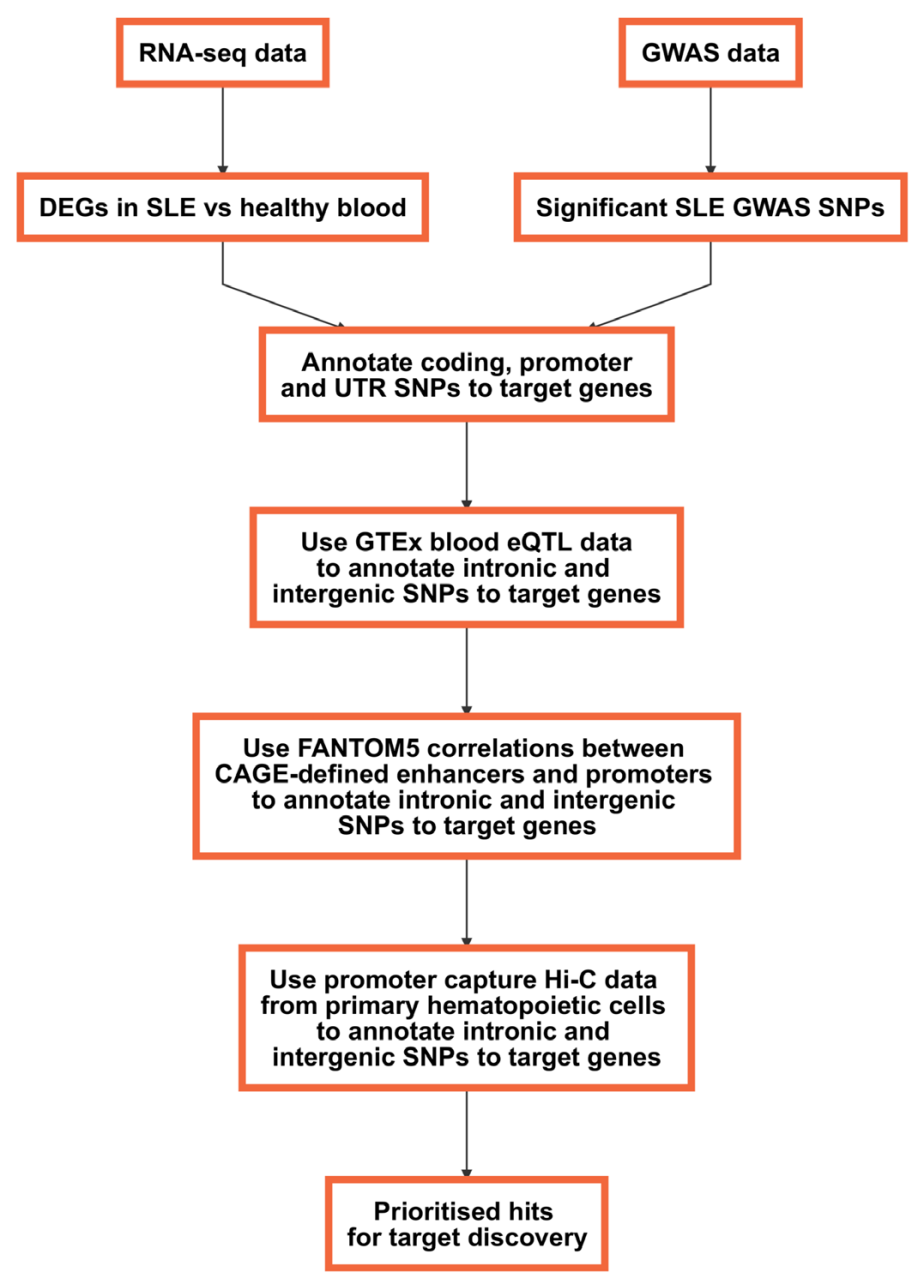
Diagram showing a schematic representation of the workflow and the steps involved.

In this workflow, we will explore how regulatory genomic data can be used to connect the genetic and transcriptional layers by providing a framework for the discovery of novel therapeutic targets. We will use eQTL data from GTEx ^10^, FANTOM5 correlations between promoters and enhancers ^17^ and promoter capture Hi-C data ^21^ to annotate significant GWAS variants to putative target genes and to prioritise genes obtained from a differential expression analysis ( Figure 1).

In this workflow, we will explore how regulatory genomic data can be used to connect the genetic and transcriptional layers by providing a framework for the discovery of novel therapeutic targets. We will use eQTL data from GTEx ^10^, FANTOM5 correlations between promoters and enhancers ^17^ and promoter capture Hi-C data ^21^ to annotate significant GWAS variants to putative target genes and to prioritise genes obtained from a differential expression analysis ( Figure 1).

## Workflow

### Install required packages

R version 3.4.2 and Bioconductor version 3.6 were used for the analysis. The code below will install all required packages and dependencies from Bioconductor and CRAN:



source("https://bioconductor.org/biocLite.R")

# uncomment the following line to install packages
#biocLite(c("clusterProfiler", "DESeq2", "GenomicFeatures", 
"GenomicInteractions", "GenomicRanges", "ggplot2", "Gviz", "gwascat", 
"InteractionSet", "recount", "pheatmap", "RColorBrewer", "rtracklayer", 
"R.utils", "splitstackshape", "VariantAnnotation"))
                    


### Gene expression data and differential gene expression analysis

We start with a common scenario: we ran a RNA-seq experiment comparing patients with a disease and healthy individuals, and would like to discover key disease genes and potential therapeutic targets by integrating genetic information in our analysis.

The RNA-seq data we will be using comes from blood of patients with systemic lupus erythematosus (SLE) and healthy controls ^24^. SLE is a chronic autoimmune disorder that can affect several organs with a significant unmet medical need ^25^. It is a complex and remarkably heterogeneous disease, in terms of both genetics and clinical manifestations ^26^. Early diagnosis and classification of SLE remain extremely challenging ^27^.

In the original study ^24^, the authors explore transcripts bound by Ro60, an RNA-binding protein against which some SLE patients produce autoantibodies. They identify Alu retroelements among these transcripts and use RNA-seq data to check their expression levels, observing that Alu elements are significantly more expressed in SLE patients, and particularly in those patients with anti-Ro antibodies and with a higher interferon signature metric (ISM).

We are going to use recount ^28^ to obtain gene-level counts:



library(recount)
# uncomment the following line to download dataset
#download_study("SRP062966") 
load(file.path("SRP062966","rse_gene.Rdata"))
rse <- scale_counts(rse_gene)
                    


Other Bioconductor packages that can be used to access data from gene expression experiments directly in R are GEOquery ^29^ and ArrayExpress ^30^.

We have 117 samples overall. This is what the matrix of counts looks like:



assay(rse)[1:3,1:3]
                    




##	              SRR2443263  SRR2443262  SRR2443261
## ENSG00000000003.14	      19	   6	      10
## ENSG00000000005.5	       0	   0	       0
## ENSG00000000419.12	     489         238	     224
                    


Each gene is a row and each sample is a column. We note that genes are annotated using the GENCODE ^31^ v25 annotation, which will be useful later on.

To check how we can split samples between cases and controls, we can have a look at the metadata contained in the characteristics column, which is a CharacterList object:



head(rse$characteristics,3)
                    




## CharacterList of length 3
## [[1]] disease status: healthy tissue: whole blood anti-ro: control ism:
control
## [[2]] disease status: healthy tissue: whole blood anti-ro: control ism:
control
## [[3]] disease status: healthy tissue: whole blood anti-ro: control ism:
control
                    


We have information about the disease status of the sample, the tissue of origin, the presence and level of anti-ro autoantibodies and the value of the ISM. However, we note that basic information such as age or gender is missing.

We can create some new columns with the available information so that they can be used for downstream analyses. We will also make sure that they are encoded as factors and that the correct reference layer is used:



# disease status
rse$disease_status <- sapply(rse$characteristics,"[",1)
rse$disease_status <- sub("disease status: ","", rse$disease_status)"systemic lupus erythematosus \\(SLE\\)","SLE",
rse$disease_status)rse$disease_status <- factor(rse$disease_status, levels = c("healthy","SLE"))
# tissue
rse$tissue <- sapply(rse$characteristics,"[",2)
rse$tissue <- sub("tissue: ","", rse$tissue)
rse$tissue <- factor(rse$tissue)
# anti-ro
rse$anti_ro <- sapply(rse$characteristics,"[",3)
rse$anti_ro <- sub("anti-ro: ","", rse$anti_ro)
rse$anti_ro <- factor(rse$anti_ro)# ism
rse$ism <- sapply(rse$characteristics,"[",4)
rse$ism <-sub("ism: ","", rse$ism)
rse$ism <- factor(rse$ism)
                    


We can check how many samples we have in each group (note that we ignore tissue as it's always whole blood):



metadata <- data.frame(disease_status = rse$disease_status, anti_ro.ism = paste(rse$anti_ro, rse$ism, sep ="."))
table(metadata)
                    




##               anti_ro.ism
## disease_status control.control high.ISM_high high.ISM_low med.ISM_high
##        healthy              18             0            0            0
##        SLE                   0            23            1           21
##               anti_ro.ism 
## disease_status med.ISM_low none.ISM_high none.ISM_low
##        healthy           0             0            0
##        SLE               2            31           21
                    


Now we are ready to perform a simple differential gene expression analysis with DESeq2 ^32^. Note that we remove genes with a low number of counts (less than 50 across all 117 samples) to speed up execution and reduce the memory footprint:



library(DESeq2)
dds <- DESeqDataSet(rse, ~ disease_status)
dds <- DESeq(dds)
dds <- dds[rowSums(counts(dds)) >= 50, ]
                    


We used an extremely simple model; in the real world we should be accounting for co-variables, potential confounders and interactions between them. For example, age and gender are usually included in this type of analysis, but we don't have access to this information for this dataset. Similarly, the value of the ISM and the presence of anti-Ro autoantibodies can't be included in the analysis due to the fact that these variables are collinear with the disease status variable (i.e.: the value of both anti_ro and ism is control for all samples with disease_status equal to healthy.) Like DESeq2, edgeR ^33^ and limma ^34^ can also deal with multiple cofactors and different experimental designs, and constitute good alternatives for performing differential expression analyses.

We can now look at the data in more detail to assess if we can observe a separation between the SLE and healthy samples and whether any batch effect is visible. We use the variance stabilising transformation (VST) ^35^ for visualisation purposes:



vsd <- vst(dds, blind = FALSE)
                    


**Figure 2.**
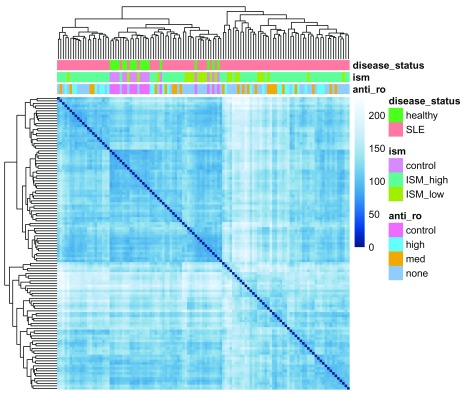
Heatmap showing Euclidean distances between samples clustered using complete linkage. Disease status and other experimental factors are visualised as column annotations.

We will use the pheatmap and RColorBrewer packages to perform hierarchical clustering of the samples ( Figure 2):



library(pheatmap)
library(RColorBrewer)
sampleDists <- dist(t(assay(vsd)))
sampleDistMatrix <- as.matrix(sampleDists)
annotation = data.frame(colData(vsd)[c("anti_ro", "ism","disease_status")],
row.names = rownames(sampleDistMatrix))
colors <- colorRampPalette(rev(brewer.pal(9,"Blues")))(255)
pheatmap(sampleDistMatrix, clustering_distance_rows = sampleDists,
clustering_distance_cols = sampleDists, clustering_method ="complete",
annotation_col = annotation, col = colors, show_rownames =FALSE,
show_colnames =FALSE, cellwidth =2, cellheight =2)
                    


While there isn't an unambiguous split between healthy and disease samples, the most distinct clusters (bottom right and top left) are entirely composed of SLE samples, with the central cluster containing all healthy samples and a number of SLE ones. The clusters don't appear to be due to the ISM or the presence of anti-Ro autoantibodies.

**Figure 3.**
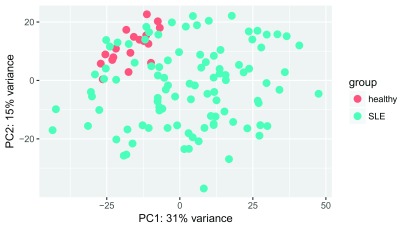
Scatter plot showing results of a PCA with samples coloured according to their disease status.

Similarly, we can perform a principal component analysis (PCA) on the most variable 500 genes ( Figure 3). Note that we load ggplot2 ^36^ to modify the look of the plot:



library(ggplot2)
plotPCA(vsd, intgroup ="disease_status") +
  coord_fixed()
                    


We can see some separation of healthy and SLE samples along both PC1 and PC2, though some SLE samples appear very similar to the healthy ones. No obvious batch effects are visible from this plot.

Next, we select genes that are differentially expressed below a 0.05 adjusted *p*-value threshold:




res <- results(dds, alpha = 0.05)
summary(res)





##
## out of 32820 with nonzero total read count
## adjusted p-value < 0.05
## LFC > 0 (up)     : 4829, 15%
## LFC < 0 (down)   : 2709, 8.3%
## outliers [1]     : 0, 0%
## low counts [2]   : 2548, 7.8%
## (mean count < 1)
## [1] see 'cooksCutoff' argument of ?results
## [2] see 'independentFiltering' argument of ?results
                    


**Figure 4.**
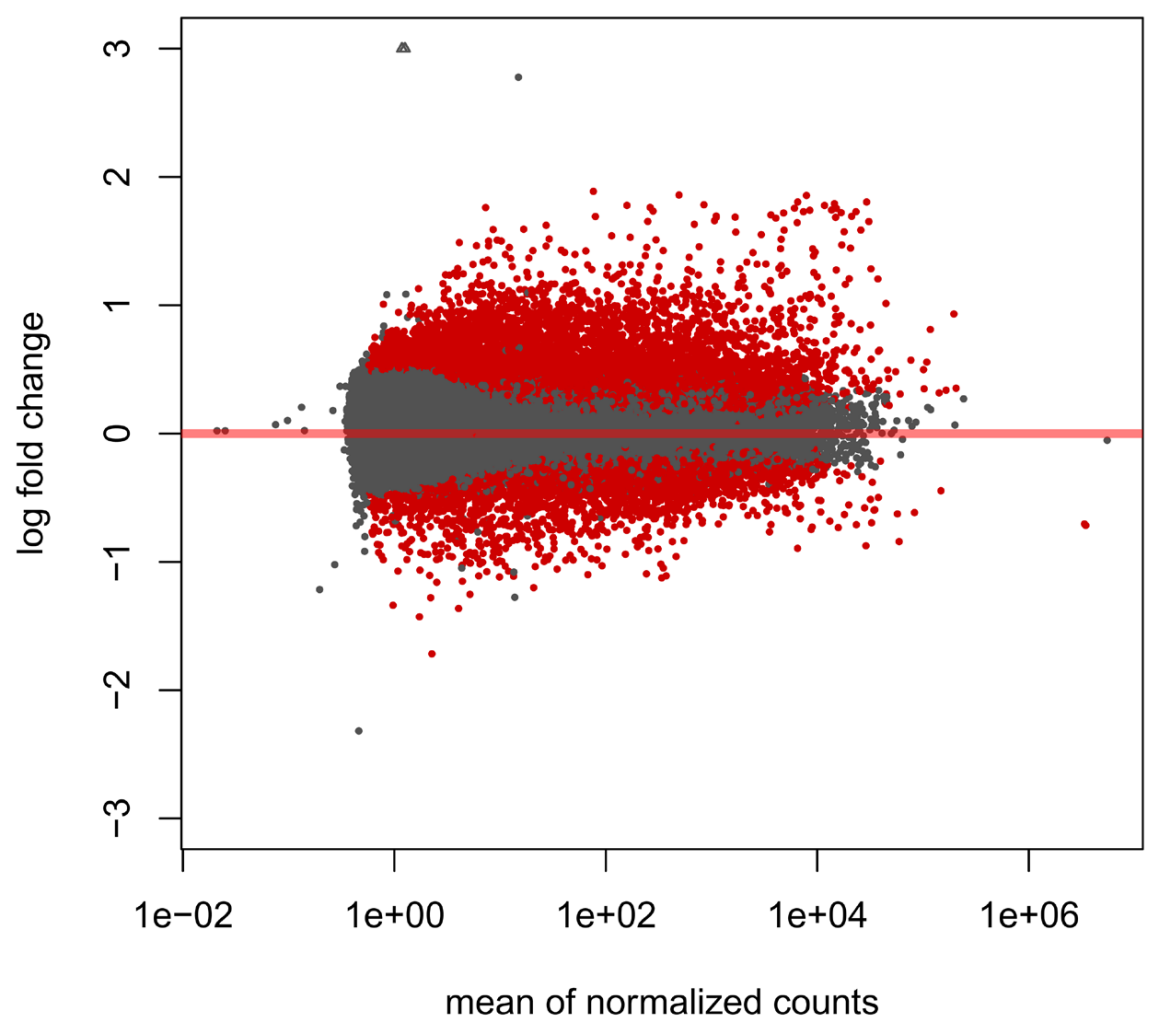
MA plot showing genes differentially expressed (red dots) in SLE patients compared to healthy patients.

We can visualise the shrunken log2 fold changes using an MA plot ( Figure 4):



res_lfc <- lfcShrink(dds, coef = 2)
plotMA(res_lfc, ylim = c(-3,3))
                    


We observe large numbers of genes differentially expressed in both directions and across a range of fold changes, though the majority of significant genes appear to be upregulated in disease.

For convenience, we will save our differentially expressed genes (DEGs) in another object and map the GENCODE gene IDs to gene symbols using the annotation in the original RangedSummarizedExperiment object



degs <- subset(res, padj <0.05)
degs <- merge(rowData(rse), as.data.frame(degs), by.x ="gene_id", by.y =
"row.names", all =FALSE)
head(degs,3)





## DataFrame with 3 rows and 9 columns
##                            gene_id bp_length symbol   baseMean
##                        <character> <integer> <list>  <numeric>
## ENSG00000000003 ENSG00000000003.14      4535 TSPAN6   8.739822
## ENSG00000000419 ENSG00000000419.12      1207   DPM1 431.485085
## ENSG00000000457 ENSG00000000457.13      6883  SCYL3 686.579323
##                 log2FoldChange      lfcSE      stat       pvalue
##                      <numeric>  <numeric> <numeric>    <numeric>
## ENSG00000000003     -0.4750382 0.18374822 -2.585267 9.730366e-03
## ENSG00000000419      0.5559772 0.10117967  5.494950 3.908216e-08
## ENSG00000000457      0.1927081 0.05928191  3.250707 1.151185e-03
##                         padj
##                    <numeric>
## ENSG00000000003 4.161281e-02
## ENSG00000000419 2.182977e-06
## ENSG00000000457 7.922475e-03



### Accessing GWAS data

The differential expression analysis resulted in several thousands of DEGs. Since we know that genes with high levels of differential expression are more likely to harbour disease-associated variants ^37^ and that therapeutic targets with genetic evidence are more likely to progress through the drug discovery pipeline ^6^, one way to prioritise them is to check which of these can be genetically linked to SLE. To get hold of relevant GWAS data, we will be using the gwascat Bioconductor package ^38^, which provides an interface to the GWAS catalog ^39^. An alternative is to use the GRASP ^40^ database with the grasp2db ^41^ package.



library(gwascat)
# uncomment the following line to download file and build the gwasloc object
all in one step
#snps <- makeCurrentGwascat()
# uncomment the following line to download file
#download.file("http://www.ebi.ac.uk/gwas/api/search/downloads/alternative",
destfile = "gwas_catalog_v1.0.1-associations_e90_r2017-12-04.tsv")
snps <- read.delim("gwas_catalog_v1.0.1-associations_e90_r2017-12-04.tsv",
check.names =FALSE, stringsAsFactors =FALSE)
snps <- gwascat:::gwdf2GRanges(snps, extractDate ="2017-12-04")
genome(snps) <-"GRCh38"
head(snps,3)
                    




## gwasloc instance with 3 records and 37 attributes per record.
## Extracted:  2017-12-04
## Genome:  GRCh38
## Excerpt:
## GRanges object with 3 ranges and 3 metadata columns:
##       seqnames                 ranges strand | DISEASE/TRAIT        SNPS
##          <Rle>              <IRanges>  <Rle> |   <character> <character>
##   [1]     chr1 [203186754, 203186754]      * | YKL-40 levels   rs4950928
##   [2]    chr13 [ 39776775,  39776775]      * |     Psoriasis   rs7993214
##   [3]    chr15 [ 78513681,  78513681]      * |   Lung cancer   rs8034191
##         P-VALUE
##       <numeric>
##   [1]     1e-13
##   [2]     2e-06
##   [3]     3e-18
##   -------
##   seqinfo: 23 sequences from GRCh38 genome; no seqlengths
                    


snps is a gwasloc object which is simply a wrapper around a GRanges object, the standard way to represent genomic ranges in Bioconductor.

We note here that the GWAS catalog uses GRCh38 coordinates, the same assembly used in the GENCODE v25 annotation. When integrating genomic datasets from different sources it is essential to ensure that the same genome assembly is used, especially because many datasets in the public domain are still using GRCh37 coordinates. As we will see below, it is possible and relatively straightforward to convert genomic coordinates between genome assemblies.

We can select only SNPs that are associated with SLE:



snps <- subsetByTraits(snps, tr ="Systemic lupus erythematosus")
                    


**Figure 5.**
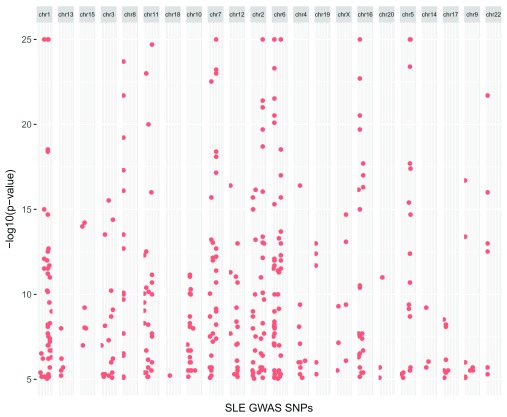
Manhattan plot showing GWAS variants significantly associated with SLE.

We can visualise these as a Manhattan plot to look at the distribution of GWAS *p*-values over chromosomes on a negative log _10_ scale ( Figure 5): Note that *p*-values lower than 1 × 10 ^-25^ are truncated in the figure:



traitsManh(gwr = snps, sel = snps, traits ="Systemic lupus erythematosus") +
  xlab("SLE GWAS SNPs") +
  ylab("-log10(p-value)") +
  theme(legend.position ="none",
        strip.text.x = element_text(size =6),
        axis.text.x = element_blank(),
        axis.ticks.x = element_blank())
                    


We observe several hits across most chromosomes, with many of them below a genome-wide significant threshold ( *p*-value < 1 × 10 ^-8^), suggesting that genetics plays an important role in the pathogenesis of SLE.

We note here that genotyping arrays typically include a very small fraction of all possible SNPs in the human genome, and there is no guarantee that the *tag* SNPs on the array are the true casual SNPs ^42^. The alleles of other SNPs can be imputed from tag SNPs thanks to the structure of linkage disequilibrium (LD) blocks present in chromosomes. Thus, when linking variants to target genes in a real-world setting, it is important to take into consideration neighbouring SNPs that are in high LD (e.g.: r ^2^ > 0.8) and inherited with the tag SNPs. Unfortunately, at the time of writing there is no straightforward way to perform this LD expansion step using R or Bioconductor packages, possibly because of the large amount of reference data required. The ldblock package ^43^ used to provide this functionality by downloading the HapMap data from the NCBI website, but the dataset was retired in 2016. At present, the best option to do this programmatically is probably to query the Ensembl REST API ^44^.

### Annotation of coding and proximal SNPs to target genes

In order to annotate these variants, we need a a TxDb object, a reference of where transcripts are located on the genome. We can build this using the GenomicFeatures ^45^ package and the GENCODE v25 gene annotation:



library(GenomicFeatures)
# uncomment the following line to download file
#download.file("ftp://ftp.sanger.ac.uk/pub/gencode/Gencode_human/release_25/gencode.v25.annotation.gff3.gz", destfile = "gencode.v25.annotation.gff3.gz")
txdb <- makeTxDbFromGFF("gencode.v25.annotation.gff3.gz")
txdb <- keepStandardChromosomes(txdb)



We also have to convert the gwasloc object into a standard GRanges object:



snps <- GRanges(snps)



Let's check if the gwasloc and TxDb object use the same notation for chromosomes:



seqlevelsStyle(snps)






## [1] "UCSC"






seqlevelsStyle(txdb)






## [1] "UCSC"



OK, they do. Now we can annotate our SNPs to genes using the VariantAnnotation ^46^ package:




library(VariantAnnotation)
snps_anno <- locateVariants(snps, txdb, AllVariants())
snps_anno <- unique(snps_anno)



We use the QUERYID column in snps_anno to recover metadata such as SNP IDs and GWAS *p*-values from the original snps object:




snps_metadata <- snps[snps_anno$QUERYID]
mcols(snps_anno) <- cbind(mcols(snps_metadata)[c("SNPS","P-VALUE")],
mcols(snps_anno))



**Figure 6.**
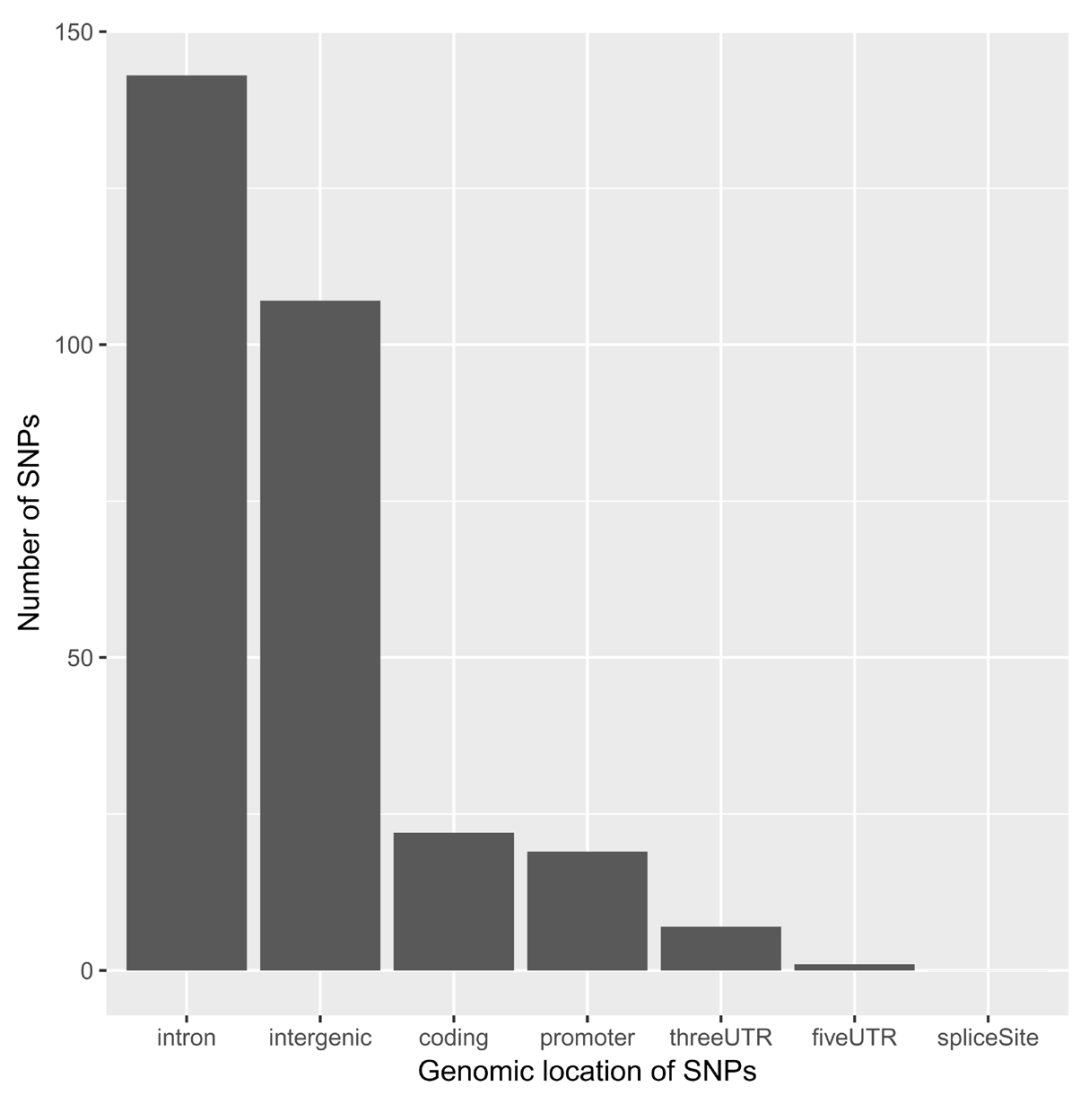
Bar plot showing genomic locations associated with SLE variants.

We can visualise where these SNPs are located ( Figure 6):




loc <- data.frame(table(snps_anno$LOCATION))
ggplot(data = loc, aes(x = reorder(Var1, -Freq), y = Freq)) +
  geom_bar(stat = "identity") +
  xlab("Genomic location of SNPs") +
  ylab("Number of SNPs")



As expected ^7^, the great majority of SNPs are located within introns and in intergenic regions. For the moment, we will focus on SNPs that are either coding or in promoter and UTR regions, as these can be assigned to target genes rather unambiguously:




snps_easy <- subset(snps_anno, LOCATION == "coding"| LOCATION == "promoter"|
LOCATION == "threeUTR"| LOCATION == "fiveUTR")
snps_easy <- as.data.frame(snps_easy)



Now we can check if any of the genes we found to be differentially expressed in SLE is also genetically associated with the disease:




snps_easy_in_degs <- merge(degs, snps_easy, by.x = "gene_id", by.y = "GENEID", all = FALSE)



We have 14 genes showing differential expression in SLE that are also genetically associated with the disease. While this is an interesting result, these hits are likely to be already well-known as potential SLE targets given their clear genetic association.

We will store essential information about these hits in a results data.frame:




prioritised_hits <- unique(data.frame(
  snp_id = snps_easy_in_degs$SNPS,
  snp_pvalue = snps_easy_in_degs$P.VALUE,
  snp_location = snps_easy_in_degs$LOCATION,
  gene_id = snps_easy_in_degs$gene_id,
  gene_symbol = snps_easy_in_degs$symbol,
  gene_pvalue = snps_easy_in_degs$padj,
  gene_log2foldchange = snps_easy_in_degs$log2FoldChange,
  method ="Direct overlap",
  row.names =NULL))
head(prioritised_hits,3)





##       snp_id snp_pvalue snp_location            gene_id gene_symbol
## 1  rs1887428      1e-06      fiveUTR ENSG00000096968.13        JAK2
## 2 rs58688157      5e-13     promoter ENSG00000099834.18       CDHR5
## 3  rs1990760      4e-08       coding  ENSG00000115267.5       IFIH1
##    gene_pvalue gene_log2foldchange         method
## 1 1.951160e-04            0.636590 Direct overlap
## 2 1.455662e-05            1.033372 Direct overlap
## 3 2.719420e-10     	     1.745324 Direct overlap



### Use of regulatory genomic data to map intronic and intergenic SNPs to target genes

But what about all the SNPs in introns and intergenic regions? Some of those might be regulatory variants affecting the expression level of their target gene(s) through a distal enhancer. Let's create a dataset of candidate regulatory SNPs that are either intronic or intergenic and remove the annotation obtained with VariantAnnotation:



snps_hard <- subset(snps_anno, LOCATION =="intron"| LOCATION ==
"intergenic", select = c("SNPS","P.VALUE","LOCATION"))
                    


***eQTL data.*** A well-established way to gain insights into target genes of regulatory SNPs is to use eQTL data, where correlations between genetic variants and expression of genes are computed across different tissues or cell types ^9^. Here, we will simply match GWAS SNPs and eQTLs according to their genomic locations, which is a rather crude way to integrate these two types of data. More robust alternatives such as PrediXcan ^47^, TWAS ^48^ and SMR ^49^ exist and should be adopted if possible. One downside of these methods is that they require subject-level or complete summary data, making them less practical in some circumstances.

We will use blood eQTL data from the GTEx consortium ^10^. To get the data, you will have to register and download the file GTEx_Analysis_v7_eQTL.tar.gz from the GTEx portal to the current working directory:



# uncomment the following line to extract the gzipped archive file
#untar("GTEx_Analysis_v7_eQTL.tar.gz")
gtex_blood <-
read.delim(gzfile("GTEx_Analysis_v7_eQTL/Whole_Blood.v7.signif_variant_gene_
pairs.txt.gz"), stringsAsFactors = FALSE)
head(gtex_blood,3)
                    




##           variant_id           gene_id tss_distance ma_samples ma_count
## 1 1_231153_CTT_C_b37 ENSG00000223972.4       219284         13       13
## 2    1_61920_G_A_b37 ENSG00000238009.2       -67303         18       20
## 3    1_64649_A_C_b37 ENSG00000238009.2       -64574         16       16
##         maf pval_nominal    slope slope_se pval_nominal_threshold
## 1 0.0191740  3.69025e-08 1.319720 0.233538            1.35366e-04
## 2 0.0281690  7.00836e-07 0.903786 0.178322            8.26088e-05
## 3 0.0220386  5.72066e-07 1.110040 0.217225            8.26088e-05
##   min_pval_nominal   pval_beta
## 1      3.69025e-08 4.67848e-05
## 2      6.50297e-10 1.11312e-06
## 3      6.50297e-10 1.11312e-06
                    


We have to extract the genomic locations of the SNPs from the IDs used by GTEx:



locs <- strsplit(gtex_blood$variant_id,"_")
gtex_blood$chr <- sapply(locs,"[",1)
gtex_blood$start <- sapply(locs,"[",2)
gtex_blood$end <- sapply(locs,"[",2)
                    


We can then convert the data.frame into a GRanges object:



gtex_blood <- makeGRangesFromDataFrame(gtex_blood, keep.extra.columns = TRUE)
                    


We also need to ensure that the chromosome notation is consistent with the previous objects:



seqlevelsStyle(gtex_blood)
                    




## [1] "NCBI"    "Ensembl"
                    




seqlevelsStyle(gtex_blood) <- "UCSC"
                    


From the publication ^10^, we know the genomic coordinates are mapped to genome reference GRCh37, so we will have to uplift them to GRCh38 using rtracklayer ^50^ and a mapping ("chain") file. The R.utils package is only required to extract the gzipped file:



library(rtracklayer)
library(R.utils)
# uncomment the following line to download file
#download.file("http://hgdownload.cse.ucsc.edu/goldenPath/hg19/liftOver/hg19To
Hg38.over.chain.gz", destfile = "hg19ToHg38.over.chain.gz")
# uncomment the following line to extract gzipped file
#gunzip("hg19ToHg38.over.chain.gz")
ch <- import.chain("hg19ToHg38.over.chain")
gtex_blood <- unlist(liftOver(gtex_blood, ch))
                    


We will use the GenomicRanges package ^45^ to compute the overlap between GWAS SNPs and blood eQTLs:



library
(GenomicRanges)
hits <- findOverlaps(snps_hard, gtex_blood)
snps_hard_in_gtex_blood = snps_hard[queryHits(hits)]
gtex_blood_with_snps_hard = gtex_blood[subjectHits(hits)]
mcols(snps_hard_in_gtex_blood) <- cbind(mcols(snps_hard_in_gtex_blood),
mcols(gtex_blood_with_snps_hard))
snps_hard_in_gtex_blood <- as.data.frame(snps_hard_in_gtex_blood)
                    


We have 59 blood eQTL variants that are associated with SLE. We can now check whether any of the genes differentially expressed in SLE is an *eGene*, a gene whose expression is influenced by an eQTL. Note that gene IDs in GTEx are mapped to GENCODE v19 ^10^, while we are using the newer v25 for the DEGs. To match the gene IDs in the two objects, we will simply strip the last bit containing the GENCODE gene version, which effectively gives us Ensembl gene IDs:



snps_hard_in_gtex_blood$ensembl_id <- sub("(ENSG[0-9]+)\\.[0-9]+", "\\1",
snps_hard_in_gtex_blood$gene_id)
degs$ensembl_id <- sub("(ENSG[0-9]+)\\.[0-9]+","\\1", degs$gene_id)
snps_hard_in_gtex_blood_in_degs <- merge(snps_hard_in_gtex_blood, degs, by = "ensembl_id", all = FALSE)
                    


We can add these 17 genes to our list:



prioritised_hits <- unique(rbind(prioritised_hits, data.frame(
  snp_id = snps_hard_in_gtex_blood_in_degs$SNPS,
  snp_pvalue = snps_hard_in_gtex_blood_in_degs$P.VALUE,
  snp_location = snps_hard_in_gtex_blood_in_degs$LOCATION,
  gene_id = snps_hard_in_gtex_blood_in_degs$gene_id.y,
  gene_symbol = snps_hard_in_gtex_blood_in_degs$symbol,
  gene_pvalue = snps_hard_in_gtex_blood_in_degs$padj,
  gene_log2foldchange = snps_hard_in_gtex_blood_in_degs$log2FoldChange,
  method ="GTEx eQTLs",
  row.names = NULL)))
                    


***FANTOM5 data.*** The FANTOM consortium profiled gene expression across a large panel of tissues and cell types using CAGE ^15, 17^. This technology allows mapping of transcription start sites and enhancer RNAs genome-wide. Correlations between these promoter and enhancer elements across a large panel of tissues and cell types can then be calculated to identify significant promoter - enhancer pairs. In turn, we will use these correlations to map distal regulatory SNPs to target genes.

Let's read in the enhancer - promoter correlation data:



# uncomment the following line to download the file
#download.file("http://enhancer.binf.ku.dk/presets/enhancer_tss_associations.bed", destfile = "enhancer_tss_associations.bed")
fantom <- read.delim("enhancer_tss_associations.bed", skip =1,
stringsAsFactors = FALSE)
head(fantom,3)
                    




##   X.chrom chromStart chromEnd
## 1    chr1     858252   861621
## 2    chr1     894178   956888
## 3    chr1     901376   956888
##                                                                  name
## 1                   chr1:858256-858648;NM_152486;SAMD11;R:0.404;FDR:0
## 2 chr1:956563-956812;NM_015658;NOC2L;R:0.202;FDR:8.01154668254404e-08
## 3     chr1:956563-956812;NM_001160184,NM_032129;PLEKHN1;R:0.422;FDR:0
##   score strand thickStart thickEnd itemRgb blockCount blockSizes
## 1   404      .     858452   858453   0,0,0          2   401,1001
## 2   202      .     956687   956688   0,0,0          2   1001,401
## 3   422      .     956687   956688   0,0,0          2   1001,401
##   chromStarts
## 1      0,2368
## 2     0,62309
## 3     0,55111
                    


Everything we need is in the fourth column, name: genomic location of the enhancer, gene identifiers, Pearson correlation coefficient and significance. We will use the splitstackshape package to parse it:



library(splitstackshape)
fantom <- as.data.frame(cSplit(fantom, splitCols = "name", sep = ";",
direction = "wide"))
                    


Now we can extract the genomic locations of the enhancers and the correlation values:



locs <- strsplit(as.character(fantom$name_1),"[:-]")
fantom$chr <- sapply(locs,"[",1)
fantom$start <- as.numeric(sapply(locs,"[",2))
fantom$end <- as.numeric(sapply(locs,"[",3))
fantom$symbol <- fantom$name_3
fantom$corr <- sub("R:","", fantom$name_4)
fantom$fdr <- sub("FDR:","", fantom$name_5)
                    


We can select only the enhancer - promoter pairs with a decent level of correlation and significance and tidy the data at the same time:



fantom <- unique(subset(fantom, corr >= 0.25& fdr <1e-5, select = c("chr",
"start","end","symbol")))
                    


Now we would like to check whether any of our candidate regulatory SNPs are falling in any of these enhancers. To do this, we have to convert the data.frame into a GRanges object and uplift the GRCh37 coordinates ^15^ to GRCh38:



fantom <- makeGRangesFromDataFrame(fantom, keep.extra.columns = TRUE)
fantom <- unlist(liftOver(fantom, ch))
                    


We can now compute the overlap between SNPs and enhancers:



hits <- findOverlaps(snps_hard, fantom)
snps_hard_in_fantom = snps_hard[queryHits(hits)]
fantom_with_snps_hard = fantom[subjectHits(hits)]
mcols(snps_hard_in_fantom) <- cbind(mcols(snps_hard_in_fantom),
mcols(fantom_with_snps_hard))
snps_hard_in_fantom <- as.data.frame(snps_hard_in_fantom)
                    


Let’s check if any of these genes is differentially expressed in our RNA-seq data:



snps_hard_in_fantom_in_degs <- merge(snps_hard_in_fantom, degs, by = "symbol",
all = FALSE)
                    


We have identified 7 genes whose putative enhancers contain SLE GWAS SNPs. Let's add these to our list:



prioritised_hits <- unique(rbind(prioritised_hits, data.frame(
  snp_id = snps_hard_in_fantom_in_degs$SNPS,
  snp_pvalue = snps_hard_in_fantom_in_degs$P.VALUE,
  snp_location = snps_hard_in_fantom_in_degs$LOCATION,
  gene_id = snps_hard_in_fantom_in_degs$gene_id,
  gene_symbol = snps_hard_in_fantom_in_degs$symbol,
  gene_pvalue = snps_hard_in_fantom_in_degs$padj,
  gene_log2foldchange = snps_hard_in_fantom_in_degs$log2FoldChange,
  method = "FANTOM5 correlations",
  row.names = NULL)))
                    


***Promoter Capture Hi-C data.*** More recently, chromatin interaction data was generated across 17 human primary blood cell types using promoter capture Hi-C ^21^. More than 30,000 promoter baits were used to capture promoter-interacting regions genome-wide, which were then mapped to enhancers based on annotation present in the Ensembl Regulatory Build ^51^. This dataset provides a valuable resource for interpreting complex genomic data, especially in the context of autoimmune diseases (and other conditions where immune cells play a role). Significant interactions between enhancers and promoters can be accessed in the supplementary data of the paper:



# uncomment the following line to download file
#download.file("http://www.cell.com/cms/attachment/2086554122/2074217047/mmc4.zip", destfile = "mmc4.zip")
# uncomment the following lines to extract zipped files
#unzip("mmc4.zip")
#unzip("DATA_S1.zip")
pchic <- read.delim("ActivePromoterEnhancerLinks.tsv", stringsAsFactors = FALSE)
head(pchic,3)
                    



##   baitChr  baitSt baitEnd baitID oeChr    oeSt   oeEnd oeID
## 1    chr1 1206873 1212438    254  chr1  943676  957199  228
## 2    chr1 1206873 1212438    254  chr1 1034268 1040208  235
## 3    chr1 1206873 1212438    254  chr1 1040208 1043143  236
##                       cellType.s.
## 1                            nCD8
## 2 nCD4,nCD8,Mac0,Mac1,Mac2,MK,Mon
## 3     nCD4,nCD8,Mac0,Mac1,Mac2,MK
##
sample.s.
## 1
C0066PH1
## 2
S007DDH2,S007G7H4,C0066PH1,S00C2FH1,S00390H1,S001MJH1,S001S7H2,S0022IH2,S00622
H1,S00BS4H1,S004BTH2,C000S5H2
## 3
S007DDH2,S007G7H4,C0066PH1,S00C2FH1,S00390H1,S001MJH1,S001S7H2,S0022IH2,S00622
H1,S00BS4H1,S004BTH2


We will use the InteractionSet package ^52^, which is specifically designed for the representation of chromatin interaction data. We start by creating a GInteractions object:



library(InteractionSet)
promoters <- GRanges(seqnames = pchic$baitChr, ranges = IRanges(start =
pchic$baitSt, end = pchic$baitEnd))
enhancers <- GRanges(seqnames = pchic$oeChr, ranges = IRanges(start =
pchic$oeSt, end = pchic$oeEnd))
pchic <- GInteractions(promoters, enhancers)
                    


As gene identifiers are not provided, we also have to map promoters to the respective genes so that we know which genes are regulated by which enhancers. We can do this by using the TxDb object we previously built to extract positions of transcription start sites (TSSs) and then add the GENCODE gene IDs as metadata to the pchic object:



tsss <- promoters(txdb, upstream = 0, downstream = 1, columns = "gene_id")
hits <- nearest(promoters, tsss)
pchic$gene_id <- unlist(tsss[hits]$gene_id)
                    


Next, we calculate the overlaps between SLE GWAS SNPs and enhancers (the *second* region of the GInteractions object) :



hits <- findOverlaps(snps_hard, pchic, use.region = "second")
snps_hard_in_pchic = snps_hard[queryHits(hits)]
pchic_with_snps_hard = pchic[subjectHits(hits)]
mcols(snps_hard_in_pchic) <- cbind(mcols(snps_hard_in_pchic),
mcols(pchic_with_snps_hard))
snps_hard_in_pchic <- as.data.frame(snps_hard_in_pchic)
                    


We check if any of these enhancers containing SLE variants are known to putatively regulate genes differentially expressed in SLE:



snps_hard_in_pchic_in_degs <- merge(snps_hard_in_pchic, degs, by = "gene_id",
all = FALSE)
                    


And finally we add these 13 genes to our list:



prioritised_hits <- unique(rbind(prioritised_hits, data.frame(
  snp_id = snps_hard_in_pchic_in_degs$SNPS,
  snp_pvalue = snps_hard_in_pchic_in_degs$P.VALUE,
  snp_location = snps_hard_in_pchic_in_degs$LOCATION,
  gene_id = snps_hard_in_pchic_in_degs$gene_id,
  gene_symbol = snps_hard_in_pchic_in_degs$symbol,
  gene_pvalue = snps_hard_in_pchic_in_degs$padj,
  gene_log2foldchange = snps_hard_in_pchic_in_degs$log2FoldChange,
  method = "Promoter capture Hi-C",
  row.names = NULL)))
                    


These are the final results of our target identification exercise. We can have a look at the most significant SNPs mapped with each of the methods:



top_prioritised_hits <- prioritised_hits[order(prioritised_hits$snp_pvalue),]
top_prioritised_hits <- split(top_prioritised_hits,
top_prioritised_hits$method)
do.call(rbind, lapply(top_prioritised_hits, head, 1))
                    




##                         snp_id  snp_pvalue   snp_location            gene_id
## Direct overlap       rs3757387       1e-48       promoter ENSG00000128604.18
## GTEx eQTLs           rs1270942      2e-165         intron ENSG00000166278.14
## FANTOM5 correlations rs1150754       6e-29         intron  ENSG00000204421.2
## Promoter capture Hi-Crs1270942      2e-165         intron  ENSG00000219797.2
##                      gene_symbol   gene_pvalue   gene_log2foldchange
## Direct overlap              IRF5  5.006707e-03             0.4041349
## GTEx eQTLs                    C2  1.625111e-03             0.9269526
## FANTOM5 correlations      LY6G6C  3.575357e-05             1.4327915
## Promoter capture Hi-C         NA  1.919459e-04             0.4556364
##                                      method
## Direct overlap               Direct overlap
## GTEx eQTLs                       GTEx eQTLs
## FANTOM5 correlations  FANTOM5  correlations
## Promoter capture Hi-C Promoter capture Hi-C



**Figure 7.**
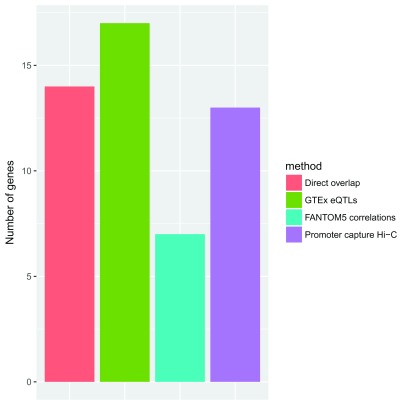
Bar plot showing number of genes identified by each variant mapping method.

We can also visualise the relative contributions from the different approaches we used ( Figure 7):



prioritised_genes <- unique(data.frame(gene_id = prioritised_hits$gene_id,
method = prioritised_hits$method))
ggplot(data = prioritised_genes, aes(x = method)) +
  geom_bar(aes(fill = method), stat ="count") +
  ylab("Number of genes") +
  theme(axis.title.x = element_blank(),
        axis.text.x = element_blank(),
        axis.ticks.x = element_blank())
                    


We observe that all methods significantly contributed to the identification of genes associated with GWAS SNPs. The majority of genes were identified through the integration of the GTEx blood eQTL data, followed by the methods based on direct overlap, promoter capture Hi-C data and FANTOM5 correlations.

### Functional analysis of prioritised hits

We will use biological processes from the Gene Ontology ^53^ and the clusterProfiler package ^54^ to functionally characterise our list of genes:



library(clusterProfiler)
prioritised_hits_ensembl_ids <- unique(sub("(ENSG[0-9]+)\\.[0-9]+","\\1",
prioritised_hits$gene_id))
all_genes_ensembl_ids <- unique(sub("(ENSG[0-9]+)\\.[0-9]+","\\1",
rownames(rse)))
gobp_enrichment <- enrichGO(prioritised_hits_ensembl_ids,
                            universe = all_genes_ensembl_ids,
                            OrgDb = org.Hs.eg.db,
                            keyType = "ENSEMBL",
                            ont = "BP",
                            pAdjustMethod = "BH",
                            pvalueCutoff = 0.05,
                            qvalueCutoff = 0.05,
                            readable = TRUE)
                    


**Figure 8.**
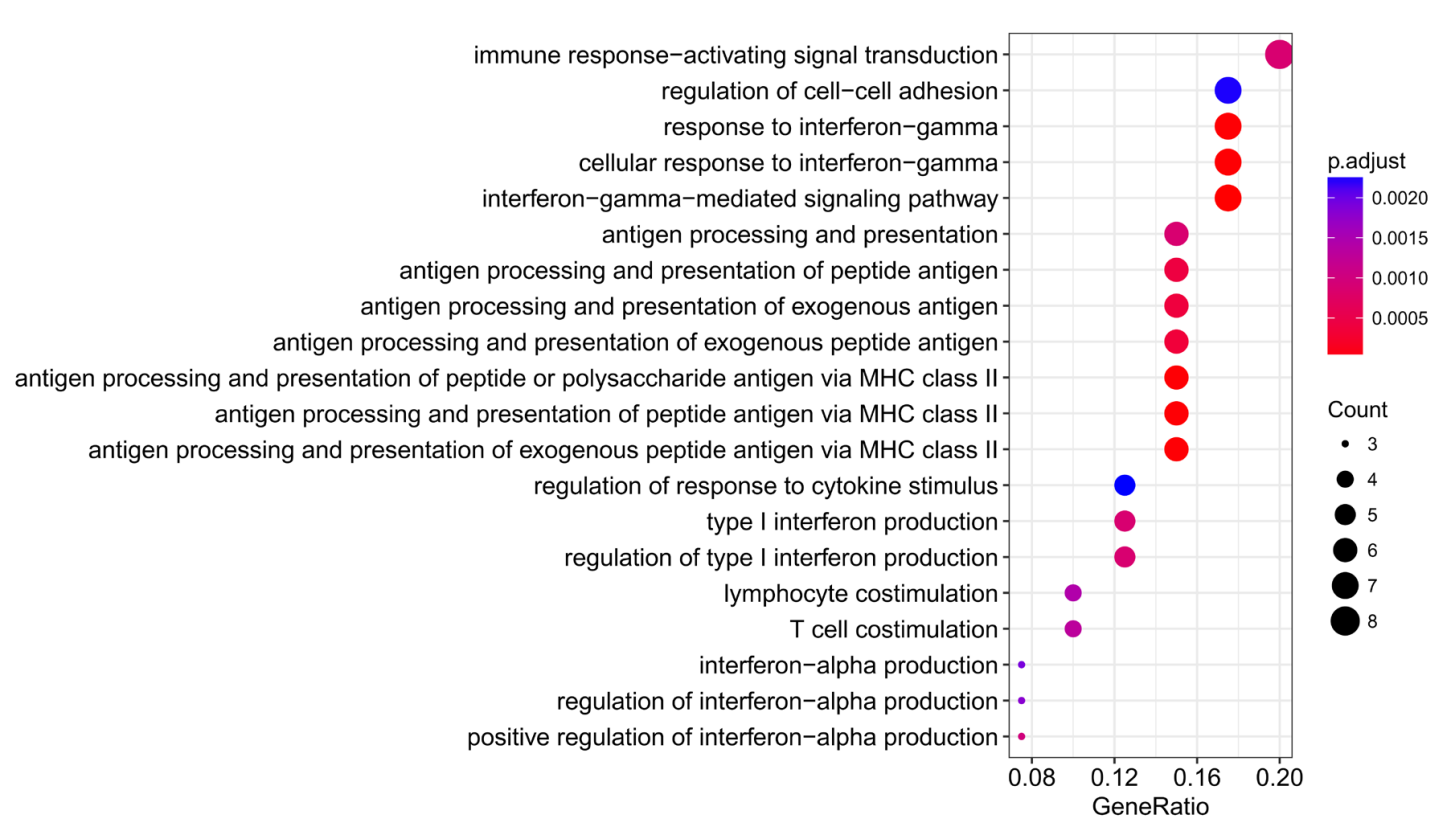
Dot plot showing enrichment of Gene Ontology biological processes for the list of prioritised genes.

We can visualise the most enriched terms ( Figure 8):



dotplot(gobp_enrichment, showCategory = 20)
                    


We observe a significant enrichment for interferon responses, antigen processing and presentation, and T cell stimulation, all processes which are well-known to play key roles in the pathogenesis of SLE ^55– 57^.

From a drug discovery perspective, JAK2 is probably the most attractive target: rs1887428 ( *p*-value = 1 × 10 ^-6^ is located in its 5' UTR and the genes is significantly upregulated in disease. Tofacitinib, a pan-JAK inhibitor, showed promising results in mouse ^58^ and is currently being tested or safety in a phase I clinical trial. We find 7 GWAS SNPs that are blood eQTLs linked to the expression of C2, a protease active in the complement signalling cascade. The most significant variant is rs1270942 ( *p*-value = 2 × 10 ^-165^) and is found in an intron of CFB, another component of the complement system. As with other autoimmune diseases, the complement plays a key role SLE in and has been investigated as a therapeutic approach ^59^. Another potentially interesting hit is TAX1BP1: rs849142 ( *p*-value = 1 × 9 ^-11^) is found within an intron of JAZF1, but can be linked to TAX1BP1 via a chromatin interaction with its promoter. TAX1BP1 inhibits TNF-induced apoptosis ^60^ and is involved in the IL1 signalling cascade ^61^, another relevant pathway in SLE that could be therapeutically targeted ^62^.

## Conclusions

In this Bioconductor workflow we have used several packages and datasets to demonstrate how regulatory genomic data can be used to annotate significant hits from GWASs and prioritise gene lists from expression studies, providing an intermediate layer connecting genetics and transcriptomics. Overall, we identified 46 SLE-associated SNPs that we mapped to 49 genes differentially expressed in SLE, using eQTL data ^10^ and enhancer - promoter relationships from CAGE ^15^ and promoter capture Hi-C experiments ^21^. These genes are involved in key inflammatory signalling pathways and some of them could develop into therapeutic targets for SLE.

The workflow also demonstrates some real-world challenges encountered when working with genomic data from different sources, such as the use of different genome assemblies and gene annotation systems, the parsing of files with custom formats into Bioconductor objects and the mapping of genomic locations to genes. While options for the visualisations of genomic data and interactions are outside the scope of this workflow, at least three good alternatives exist in Bioconductor: ggbio ^63^, Sushi ^64^ and Gviz ^65^ coupled with the GenomicInteractions package ^66^. We refer the reader to these publications and package vignettes for examples.

As the sample size and power of GWASs and gene expression studies continue to increase, it will become more and more challenging to identify truly significant hits and interpret them. The use of regulatory genomics data as presented here can be an important tool to gain insights into large biomedical datasets and help in the identification of biomarkers and therapeutic targets.

## Data and software availability

Download links for all datasets are part of the workflow. Software packages required to reproduce the analysis can be installed as part of the workflow. Source code is available at: https://github.com/enricoferrero/bioconductor-regulatory-genomics-workflow. Archived source code as at the time of publication is available at: https://doi.org/10.5281/zenodo.1154124 ^67^.

License: CC-BY 4.0
