# Supplementary material for: Using regulatory genomics data to interpret the function of disease variants and prioritise genes from expression studies
Source: F1000Res. 2018 Feb 23;7:121. Originally published 2018 Jan 29. [Version 2] doi: 10.12688/f1000research.13577.2 (PMC5850119; doi:10.12688/f1000research.13577.2)
Supplement: Peer review history

# Peer review: Referee response for version 2

**DOI:** 10.5256/f1000research.15281.r31142

**Published:** 2018-03-09

**Authors:** Vincent J. Carey

**Affiliations:** 1 Departments of Medicine, Brigham and Women's Hospital and Harvard Medical School, Boston, MA, USA

I have read this submission. I believe that I have an appropriate level of expertise to confirm that it is of an acceptable scientific standard.

# Peer review: Referee response for version 2

**DOI:** 10.5256/f1000research.15281.r31141

**Published:** 2018-03-05

**Authors:** Aaron T. L. Lun

**Affiliations:** 1 CRUK (Cancer Research UK) Cambridge Institute, University of Cambridge, Cambridge, UK

- There are some sentences where the text says "7 genes", or "13 genes".

 While this is fine, it is good practice to print "nrow(prioritised_hits)" or similar to show to readers that, yes, there are indeed the stated number of genes.

 - My suggestion for "linkOverlaps" was to do something like:

 linked <- linkOverlaps(pchic, snps_hard, tsss, use.region="same")

 ... which would give you the physical interactions between the SNPs

 (linked$subject1) and the TSS's (linked$subject2). I only mention this for future reference, as this may be more convenient in some settings.

I have read this submission. I believe that I have an appropriate level of expertise to confirm that it is of an acceptable scientific standard.

# Peer review: Referee response for version 1

**DOI:** 10.5256/f1000research.14748.r30353

**Published:** 2018-02-07

**Authors:** Vincent J. Carey

**Affiliations:** 1 Departments of Medicine, Brigham and Women's Hospital and Harvard Medical School, Boston, MA, USA

I want to preface this long review with some very broad comments. I think this undertaking is very worthwhile from several perspectives. Bioconductor is used along various avenues to create a unifiable analytic process from very diverse data resources: state-of-the-art transcriptomics from recount, **current** GWAS catalog from EMBL/EBI, variant annotation for SLE GWAS hits from the eponymous package using GENCODE for gene models, eQTL data from GTEx, enhancer annotation from FANTOM5, and promoter capture data whose origins could be better described. This is a *tour de force *but I feel it should be communicated more clearly and executed more cleanly. The paper is full of "dumps" of show events for R objects that impede the narrative flow drastically. A diagram that shows how the various resources combine in a scientifically coherent way would be a huge step forward for the paper and for practitioners. More reckoning of limitations that arise from complexity is also in order. eQTLs are far from simple, and should not be used as 'lists'. Enhancer and promoter 'lists' also need to be used with care.

 What then about this paper? It shows the resources and it shows **a path**. Isn't that enough? I don't think so. If Bioconductor and online publication make it **easier** to do and to publish complex analyses, then the presentation should be of at least as high a quality as we find in articles that are behind paywalls. In this case I feel the quality would be improved through condensation. The object dumps should be removed and replaced by meaningful tabulations and diagrams. The big picture should be stated more clearly and concisely. The limitations should also be discussed clearly. I would love to see a small set of functions that carry out the salient operations chained together to produce the solution.

 Then, given the programmatic compactness, we can discuss how to evaluate the robustness of the results of the analysis by carrying out **sensitivity analysis**. In particular, it would be great to see how the different elements of the system contribute to the ultimate enumeration of targets.

 ---

 The premise of this article is that "therapeutic targets with a genetic link to the disease under investigation are more likely to progress through the drug discovery pipeline". GWAS, PheWAS, eQTLs, epigenomic roadmap projects, and other general studies of gene regulation should be harvested to improve capacity to define genetic and genomic origins of disease, with an aim to fostering design of treatments that are focused on the molecular events underlying the disease process. The introduction concludes with mention of STOPGAP, and POSTGAP, and INFERNO, but it is not clear whether the paper is intended to describe how content of STOPGAP is developed from basic data resources like those readily available to Bioconductor users. I feel that the introduction, though well-referenced, is too long and does not clearly state the paper's main goal.

 There is no discussion of the experimental design underlying the RNA-seq study. Presumably the data were generated from this component of ref 28:

 "Finally, we tested the levels of Alu transcripts in blood cells of SLE patients and controls( 22) using RNAseq (99 active SLE, 18 healthy controls; Fig. S12). RNA-seq reads mapping to Alu elements were found at significantly higher levels in SLE subjects than controls (p=6.5E-6), Fig. 4E). Hierarchical clustering of the most highly expressed Alu RNAs ( Fig. S13) segregated Interferon Signature Metric (ISM)-high SLE subjects from control and ISM-low patients"....

 There is no discussion of heterogeneity of SLE or the difficulty of learning from a collection of 18 cases. A reference to https://www.ncbi.nlm.nih.gov/pubmed/25102991 may be in order. 

 Even though online publications are often free from page count limitations, entirely too much space is consumed by long row-broken R print events. On the one hand the recoding of SRA annotation on phenotype is important and should be exposed, on the other hand, the author could carry out the recoding programmatically in a well-parameterized function and simply update the key object by applying this function. The function can go in a package related to the paper/workflow. Instead of printing out a dataframe on p.7, it would be much better to have a contingency table showing the final layout of case and control characteristics.

 p.7 "For simplicity, we select the first 18 (healthy) and the last 18 (SLE) samples from the original RangedSummarizedExperiment object". Is this essential to the performance of the workflow? Would a more systematic matching be possible? What kind of "simplicity" does this arbitrary selection create? I understand that the main purpose of the paper is to illustrate a process, but if this thinning of the data

 is not essential to the illustration, why do it?

 p. 8: "Note that we used an extremely simple model; in the real world you will probably need to account for co-variables, potential confounders and interactions between them. edgeR and limma are good alternatives to DESEq2 for performing differential expression analyses." This suggests that you can't adjust for confounders in DESeq2, is this so? Did you not have access to any relevant cofactors in the SLE data?

 p. 9: You are really using 59000 genes after vst to do exploratory visualization of SLE vs control expression patterns? Would gene filtering be helpful? Is there any chance of batch effect or other

 surrogate variable effect that should be assessed prior to such presentations?

 By page 12 we have completed a relatively elementary differential expression analysis. It seems to me that the length of this part of the process is excessive, because the real interest is in learning about regulatory elements from other resources.

 At this point I hope I have made clear how I think the rest of the paper should be revised to make its points more effectively.

I have read this submission. I believe that I have an appropriate level of expertise to confirm that it is of an acceptable scientific standard, however I have significant reservations, as outlined above.

# Response

**Published:** 2018-02-14

**Authors:** Enrico Ferrero

**Affiliations:** GlaxoSmithKline, UK

Vincent, many thanks for reviewing my paper in depth.

 In response to your comments (please note that I took the liberty to format some of your points and omit some parts for readability):

 - [...] I think this undertaking is very worthwhile from several perspectives [..] and promoter capture data whose origins could be better described.

 --> Promoter capture Hi-C is indeed briefly introduced as a technique in the introduction. I have now added some more context on the Javierre et al., 2016 dataset and emphasised its relevance for this workflow at the beginning of the "Promoter capture Hi-C data" subsection.

 - [...] The paper is full of "dumps" of show events for R objects that impede the narrative flow drastically.

 --> I didn't realise how annoying this was until you mentioned it. I removed the great majority of dumps, leaving only a few to document the structure of datasets just imported or very final objects. For all dumps, I also ensured that a minimal amount of rows were printed.

 - A diagram that shows how the various resources combine in a scientifically coherent way would be a huge step forward for the paper and for practitioners.

 --> I included a diagram providing a schematic overview of the workflow as figure 1 and referenced it in the last paragraph of the introduction. Please note that the diagram is created in R with the DiagrammeR package but the code is hidden as it is not strictly relevant for the purposes of the workflow.

 - More reckoning of limitations that arise from complexity is also in order. eQTLs are far from simple, and should not be used as 'lists'. Enhancer and promoter 'lists' also need to be used with care.

 --> I added a few sentences at the beginning of the "eQTL data" subsection cautioning on the complexity of GWAS/eQTL integration and provided a short overview of available alternatives which are more methodologically robust.

 - [...] In particular, it would be great to see how the different elements of the system contribute to the ultimate enumeration of targets.

 --> I added figure 7 to show the relative contributions of the different approaches to the final results.

 - [...] The introduction concludes with mention of STOPGAP, and POSTGAP, and INFERNO, but it is not clear whether the paper is intended to describe how content of STOPGAP is developed from basic data resources like those readily available to Bioconductor users.

 --> I expanded that paragraph to provide more context on STOPGAP, POSTGAP and INFERNO and to clarify the intent of mentioning those resources in the introduction.

 - I feel that the introduction, though well-referenced, is too long and does not clearly state the paper's main goal.

 --> I shortened the introduction by removing the paragraph about GWAS and PheWAS and by removing or shortening several other sentences. I added a short, final paragraph stating more clearly the main goals of the workflow.

 - There is no discussion of the experimental design underlying the RNA-seq study. Presumably the data were generated from this component of ref 28: [...]

 --> That's correct. I added more context on the original study, including an overview of the experimental design, in the third paragraph of the "Gene expression data and differential gene expression analysis" section.

 - There is no discussion of heterogeneity of SLE or the difficulty of learning from a collection of 18 cases. A reference to https://www.ncbi.nlm.nih.gov/pubmed/25102991 may be in order.

 --> I addressed this point with a better introduction to SLE and its heterogeneity in the second paragraph of the "Gene expression data and differential gene expression analysis" section.

 - [...] Instead of printing out a dataframe on p.7, it would be much better to have a contingency table showing the final layout of case and control characteristics.

 --> I agree this is a more effective way to summarise the data. I removed the dataframe printing and included a contingency table showing features of case and control samples.

 - p.7 "For simplicity, we select the first 18 (healthy) and the last 18 (SLE) samples from the original RangedSummarizedExperiment object". Is this essential to the performance of the workflow? Would a more systematic matching be possible? What kind of "simplicity" does this arbitrary selection create? I understand that the main purpose of the paper is to illustrate a process, but if this thinning of the data is not essential to the illustration, why do it?

 --> Indeed, this was mostly done to speed up execution while compiling the document. I removed that chunk and all 117 samples are now used in the analysis.

 - p. 8: "Note that we used an extremely simple model; in the real world you will probably need to account for co-variables, potential confounders and interactions between them. edgeR and limma are good alternatives to DESEq2 for performing differential expression analyses." This suggests that you can't adjust for confounders in DESeq2, is this so? Did you not have access to any relevant cofactors in the SLE data?

 --> I reworded that sentence to clarify that DESEq2 is equivalent to edgeR and limma when it comes to multiple cofactors in the model. I also included a better description of the metadata available for this dataset and explained why it is not possible to include demographic statistics (unavailable) or other experimental factors (collinear with disease status) in the model.

 - p. 9: You are really using 59000 genes after vst to do exploratory visualization of SLE vs control expression patterns? Would gene filtering be helpful? Is there any chance of batch effect or other

 surrogate variable effect that should be assessed prior to such presentations?

 --> I have now applied a simple filter to remove genes with extremely low counts directly on the dds object and ahead of VST, as documented in the DESeq2 vignette [1] and the Bioconductor RNA-seq workflow [2]. This reduces the number of genes considerably, helping to speed up code execution too. I also clarified in the "Gene expression data and differential gene expression analysis" section that one of the aims of the hierarchical clustering and PCA in figure 2 and 3 is indeed to assess presence of batch effects or surrogate variables. Note that all available experimental variables are now included as annotation in the heatmap in figure 1.

 - By page 12 we have completed a relatively elementary differential expression analysis. It seems to me that the length of this part of the process is excessive, because the real interest is in learning about regulatory elements from other resources.

 --> The "Gene expression data and differential gene expression analysis" has now been considerably condensed by removing superfluous object dumps, merging code chunks and reducing the text to a minimum. One could go as far as removing the exploratory data analysis and figures, but I'd rather keep them to provide some context and a minimal differential expression analysis to be used as the starting point for the integration of the GWAS data.

 - At this point I hope I have made clear how I think the rest of the paper should be revised to make its points more effectively.

 --> Indeed. The workflow was largely redacted, condensed and improved by limiting R object dumps, providing more context on the features of the datasets used and more insights into the methodology and results of the analysis through the use of visualisations and data summaries.

 [1] https://bioconductor.org/packages/release/bioc/vignettes/DESeq2/inst/doc/DESeq2.html

 [2] https://www.bioconductor.org/help/workflows/rnaseqGene/

# Peer review: Referee response for version 1

**DOI:** 10.5256/f1000research.14748.r30355

**Published:** 2018-02-05

**Authors:** Aaron T. L. Lun

**Affiliations:** 1 CRUK (Cancer Research UK) Cambridge Institute, University of Cambridge, Cambridge, UK

This workflow is clear, well-written and makes good use of existing resources and Bioconductor software. It addresses an interesting problem in the integration of RNA-seq, GWAS, eQTL and Hi-C data for causal gene discovery in disease contexts. However, it would benefit from some more elaboration in certain sections. I have listed my comments below in more detail, ordered by the location in the workflow they refer to. For most part, I believe they are easily addressed.

 - The final paragraph of the introduction seems out of place; I do not see any reference to POSTGAP, STOPGAP or INFERNO anywhere else in the article. Was the workflow presented here used to identify the candidate genes in these resources?

 - A more comprehensive description of the SLE data set, and the motivation behind using it, would be helpful.

 - There seems to be a typo when loading the SRP062966 dataset; it should be **load(file.path("SRP062966", "rse_gene.Rdata"))**, at least on my machine.

 - I don't see why it's desirable to call **scale_counts()**. Major DE analysis frameworks are easily capable of handling differences in library sizes. Direct scaling would actually be detrimental to NB models like *edgeR* and *DESeq2*, as it distorts the mean-variance relationship. In particular, scaled counts can have sub-Poisson variation, which cannot be handled by NB models. It seems better to call **read_counts()** to obtain the gene-level read counts.

 - **rse$FIELD** can be used instead rather than **colData(rse)$FIELD**, which may simplify the code.

 - Some explanation of the other factors (anti-rho, ISM) would be helpful, given that the effort has already been taken to define them.

 - The simplicity of the model used in the DE analysis is probably unhelpful in the context described in the workflow. I would like to see more elaboration on how to handle batch effects and other confounding factors that are almost definitely present in large-scale studies. For example, what happens to the DE genes when additional explanatory factors are added to the model, e.g., anti-rho or ism status? Presumably age and sex are also relevant factors, if that information is available in the data set.

 - Generally, some of the plots could be accompanied by more commentary in text, explaining how to interprete the plot. For example, the MA plot in Figure 3 shows that DE genes are detected in both directions, at a range of abundances. It would be similarly useful to have text for the heatmap in Figure 1 and the Manhattan plot in Figure 4, among others.

 - LD expansion seems like quite an important step, especially when SNPs are being linked to genes based on overlaps to promoters/UTRs. If the LD blocks are large, expansion would result in many more potential causal SNPs and a greater number of overlaps (and thus candidate genes). While I appreciate the attempt to simplify the workflow, skipping this step seems like it would unnecessarily reduce the number of candidate genes.

 - **snps** seems to have GRCh38 coordinates. Is this also the case for GENCODE 25? It would be helpful to have a cautionary note regarding the need to make sure the same version of the genome is used throughout a workflow. I recognise that this is mentioned later when **liftOver()** is used, but it is better to be explicit about this where possible.

 - Oscillating between **head()** and **tail()** to preview the dataset is unhelpful and confusing.

 - While I don't expect a thorough examination of the set of (7 easy, 4 hard, 3 via Hi-C) candidate genes for SLE, some discussion of the biological significance of the detected genes would be appreciated. It would provide a high-level validation of the workflow and link it back to the drug discovery context.

 - For the promoter Hi-C section, you could consider using the **linkOverlaps()** method in the *InteractionSet* package, to link SNPs to gene promoters via the identified Hi-C interactions. This might be simpler than the current code, and possibly faster; the **nearest()** step in particular takes quite a long time.

I have read this submission. I believe that I have an appropriate level of expertise to confirm that it is of an acceptable scientific standard, however I have significant reservations, as outlined above.

# Response

**Published:** 2018-02-14

**Authors:** Enrico Ferrero

**Affiliations:** GlaxoSmithKline, UK

Aaron, many thanks for reviewing my paper in depth.

 In response to your comments:

 - The final paragraph of the introduction seems out of place; I do not see any reference to POSTGAP, STOPGAP or INFERNO anywhere else in the article. Was the workflow presented here used to identify the candidate genes in these resources?

 --> I expanded the paragraph to provide more context on STOPGAP, POSTGAP and INFERNO and to clarify why they are mentioned in the introduction but not used in the actual workflow.

 - A more comprehensive description of the SLE data set, and the motivation behind using it, would be helpful.

 --> More background and details on the dataset have been added in the second and third paragraph of the section "Gene expression data and differential gene expression analysis".

 - There seems to be a typo when loading the SRP062966 dataset; it should be load(file.path("SRP062966", "rse_gene.Rdata")), at least on my machine.

 --> Fixed, thanks.

 - I don't see why it's desirable to call scale_counts(). Major DE analysis frameworks are easily capable of handling differences in library sizes. Direct scaling would actually be detrimental to NB models like edgeR and DESeq2, as it distorts the mean-variance relationship. In particular, scaled counts can have sub-Poisson variation, which cannot be handled by NB models. It seems better to call read_counts() to obtain the gene-level read counts.

 --> For this section, I followed the recount quick start guide [1] and workflow [2]. Both show scaling of the counts with scale_counts() before feeding these to DESeq2. I tried switching to read_counts() but, somewhat counter-intuitively, the function returns values with decimal numbers, which in turn causes an error ("some values in assay are not integers") when calling the DESeqDataSet() function. As both scale_counts() and read_counts() seem to be acceptable, but the first one is the preferred approach by the recount developers, I switched back to scale_counts() after encountering the DESeq2 error above. The other option would have been to manually round the numbers returned by read_counts() but that seemed more questionable to me than scaling them.

 - rse$FIELD can be used instead rather than colData(rse)$FIELD, which may simplify the code.

 --> Simplified, thanks.

 - Some explanation of the other factors (anti-rho, ISM) would be helpful, given that the effort has already been taken to define them.

 --> I added context for these experimental factors in the third paragraph of the "Gene expression data and differential gene expression analysis" section and after printing the rse$characteristics object is printed.

 - The simplicity of the model used in the DE analysis is probably unhelpful in the context described in the workflow. I would like to see more elaboration on how to handle batch effects and other confounding factors that are almost definitely present in large-scale studies. For example, what happens to the DE genes when additional explanatory factors are added to the model, e.g., anti-rho or ism status? Presumably age and sex are also relevant factors, if that information is available in the data set.

 --> I agree this is not ideal, but there are good reasons why other factors are not included. First, age, gender or other demographics are not available for this dataset. Second, the ISM and anti-Ro factors are disease characteristics and are obviously only measured on the SLE patients (and not on the healthy ones). If either or both of those factors are included in the model, you get the classic "model matrix is not full rank" error [3] because they are both collinear with the disease status (all healthy samples are "control" for both anti-Ro and ISM). I've been more explicit about these shortcomings in the paragraph following the code chunk where the model is built.

 - Generally, some of the plots could be accompanied by more commentary in text, explaining how to interprete the plot. For example, the MA plot in Figure 3 shows that DE genes are detected in both directions, at a range of abundances. It would be similarly useful to have text for the heatmap in Figure 1 and the Manhattan plot in Figure 4, among others.

 --> I expanded the main text and legends for figures 2, 4 and 5 (previously 1, 3 and 4) to include a better description and explanation of the plots. I believe figures 3 and 6 (previously 2 and 5) were already adequately described. I also added 3 new figures (1, 7 and 8) to clarify the steps involved in the workflow and to provide a more in-depth understanding of the final results.

 - LD expansion seems like quite an important step, especially when SNPs are being linked to genes based on overlaps to promoters/UTRs. If the LD blocks are large, expansion would result in many more potential causal SNPs and a greater number of overlaps (and thus candidate genes). While I appreciate the attempt to simplify the workflow, skipping this step seems like it would unnecessarily reduce the number of candidate genes.

 --> Unfortunately I can't come up with a good way to perform this step in R as part of the workflow at present. The ldblock package hasn't been updated in a while and its functions rely on downloading the HapMap data from the NCBI website, which was retired in 2016 and is no longer available for download [4]. Even if it was still available, it would require downloading several GBs of data, one chromosome at a time. The previously referenced trio package uses data structures specific to case - parent trio studies which are not compatible with the use case presented in the workflow and are not designed for hundreds of SNPs, and was thus removed. The Ensembl LD Calculator is a web UI with a limit of 20 SNPs per query that can't be integrated in a programmatic workflow, so it was removed too. I guess the Ensembl REST API could be an option, but it would require introducing a few new libraries and a considerable amount of code to interact with the API and parse its output into R/Bioconductor objects, with the risk of distracting the reader from the main purpose of this (Bioconductor) workflow. It would also require performing several hundreds queries in a for loop making compilation of the document extremely long and following the workflow impractical. I modified the text in the manuscript to communicate more clearly the reasons for skipping this step. If you have other suggestions on how to do this, I would be happy to consider them.

 - snps seems to have GRCh38 coordinates. Is this also the case for GENCODE 25? It would be helpful to have a cautionary note regarding the need to make sure the same version of the genome is used throughout a workflow. I recognise that this is mentioned later when liftOver() is used, but it is better to be explicit about this where possible.

 --> I added a clarification and a warning about this in the third paragraph of the "Accessing GWAS data" section, after importing the GWAS data.

 - Oscillating between head() and tail() to preview the dataset is unhelpful and confusing.

 --> I removed all instances of tail() and replaced them with head().

 - While I don't expect a thorough examination of the set of (7 easy, 4 hard, 3 via Hi-C) candidate genes for SLE, some discussion of the biological significance of the detected genes would be appreciated. It would provide a high-level validation of the workflow and link it back to the drug discovery context.

 --> I added a new section "Functional analysis of prioritised hits" (and a new figure, 8) where I describe the biological significance and functional relevance of the results, while also discussing some of the hits in more detail from a drug discovery perspective.

 - For the promoter Hi-C section, you could consider using the linkOverlaps() method in the InteractionSet package, to link SNPs to gene promoters via the identified Hi-C interactions. This might be simpler than the current code, and possibly faster; the nearest() step in particular takes quite a long time.

 --> Thanks, I had heard of the InteractionSet package but hadn't used it before. I agree it's better to represent the promoter capture Hi-C data in this native structure. I still had to use the nearest() function (which executes almost instantaneously on my laptop) to map promoters to gene IDs though. Also, note that I didn't need the linkOverlaps() function in the end and simply used findOverlaps(..., use.region = "second") instead.

 [1] https://bioconductor.org/packages/3.7/bioc/vignettes/recount/inst/doc/recount-quickstart.html

 [2] https://f1000research.com/articles/6-1558/v1

 [3] http://seqanswers.com/forums/showthread.php?t=33032

 [4] https://www.ncbi.nlm.nih.gov/variation/news/NCBI_retiring_HapMap/
